# Inborn Errors of Metabolism and Epilepsy: Current Understanding, Diagnosis, and Treatment Approaches

**DOI:** 10.3390/ijms18071384

**Published:** 2017-07-02

**Authors:** Suvasini Sharma, Asuri N. Prasad

**Affiliations:** 1 Department of Pediatrics, Lady Hardinge Medical College, New Delhi 110001, India; sharma.suvasini@gmail.com; 2 Department of Pediatrics and Clinical Neurological Sciences, Schulich School of Medicine and Dentistry, Children’s Hospital of Western Ontario and London Health Sciences Centre, London, ON N6A5W9, Canada

**Keywords:** inborn errors of metabolism, epilepsy, seizures, pyridoxine dependent epilepsy, glucose transporter defect, genetic, biochemical testing

## Abstract

Inborn errors of metabolism (IEM) are a rare cause of epilepsy, but seizures and epilepsy are frequently encountered in patients with IEM. Since these disorders are related to inherited enzyme deficiencies with resulting effects on metabolic/biochemical pathways, the term “metabolic epilepsy” can be used to include these conditions. These epilepsies can present across the life span, and share features of refractoriness to anti-epileptic drugs, and are often associated with co-morbid developmental delay/regression, intellectual, and behavioral impairments. Some of these disorders are amenable to specific treatment interventions; hence timely and appropriate diagnosis is critical to improve outcomes. In this review, we discuss those disorders in which epilepsy is a dominant feature and present an approach to the clinical recognition, diagnosis, and management of these disorders, with a greater focus on primarily treatable conditions. Finally, we propose a tiered approach that will permit a clinician to systematically investigate, identify, and treat these rare disorders.

## 1. Introduction

Inborn errors of metabolism (IEM) are the consequence of genetic defects that lead to a metabolic block in a biochemical pathway critical to cellular function, targeting many organs including the brain. The resultant biochemical perturbation results in neuronal dysfunction and the development of epileptogenesis through complex cellular mechanisms that may be specific to each disorder. Though IEM are a relatively infrequent cause of epilepsy, epileptic seizures are a common feature in several IEM. Epilepsies associated with IEM share certain features [1]. An early age of presentation, co-morbid developmental delay/regression, and resistance to conventional antiepileptic drug therapy are common to many of these disorders. The yield from genetic testing in the present context in patients with epileptic encephalopathies is about 7% in a recent series, and for treatable conditions about 4% [2].

In this review, we discuss those disorders in which epilepsy is a predominant manifestation. In addition to presenting an approach to the clinical recognition, diagnosis, and management of these disorders, the discussion will primarily consider treatable conditions.

Readers who are not familiar with the terminology used to describe seizures and epilepsy syndromes are directed to the following ILAE website that has explanatory material available on open access. URL: http://www.ilae.org/Visitors/Centre/Definition_Class.cfm.

## 2. Pathophysiology of Epilepsy in Inborn Errors of Metabolism (IEM)

The different types or forms of epilepsy that are associated with perturbations in metabolic pathways usually do not exhibit pathway specificity. Epilepsies associated with IEM frequently display age-dependent clinical presentation in terms of semiology and electroclinical features [3]. This age-dependent presentation is presumably related to the sequential development of excitatory and inhibitory pathways in the neonatal brain [1]. γ-aminobutyric acid (GABA) functions initially as an excitatory neurotransmitter in the premature infant and a developmental switch changes its role to inhibition closer to term. This switch occurs as a result of the maturation of the cation-chloride cotransporter (KCC2) [1,4]. Subsequently, there is a surge of glutamatergic-related excitatory connections, which in turn lead to a developmental phase during which the immature brain favors excitability. The immaturity of inhibitory systems during early brain development and their dysregulation under metabolic dysfunction play a major role in neonates by lowering the seizure threshold and creating an epileptogenic milieu [4,5].

Many IEM interfere with important functions of brain metabolism such as the transport and utilization of energy substrates, the production of energy-rich phosphates, the metabolic coupling between neurons and astrocytes, the neurotransmitter signaling pathways, the autoregulation of cerebral blood flow, and the transport of substrates across the blood/brain barrier [6]. In some IEM, accumulating compounds may cause direct neurotoxicity, and certain triggers, such as fever or intercurrent infections, may precipitate seizures and encephalopathy. In these disorders, it is believed that symptoms remain latent until the accumulation of toxic products is sufficient to interfere with cell functions, as in urea cycle disorders and organic acidurias [1]. Primary or secondary disturbances in the neurotransmitter pathways with excess of excitation or lack of inhibition in the immature brain can also enhance seizure activity. Other mechanisms include disturbances in neuronal membrane permeability (e.g., holocarboxylase synthetase deficiency), substrate deficiency (e.g., serine deficiency), and impaired metalation and transport (e.g., Menke’s disease) [6]. Finally, some IEM may be associated with malformation of cortical development that may be associated with epilepsy e.g., peroxisomal disorders, pyruvate dehydrogenase deficiency, Smith–Lemli–Opitz syndrome, and congenital disorders of glycosylation.

## 3. IEM and Epileptic Encephalopathy

Epileptic encephalopathy(EE) refers to a state where continuous and persistent epileptiform activity leads to cognitive and behavioral impairments that tend to be of a severity well beyond that expected to be caused by the underlying disease pathology alone, and is usually associated with progressive loss of cerebral function [6]. The causes are variable and include structural brain malformations, sequelae of perinatal brain injury, intrauterine infections, neurocutaneous disorders, chromosomal disorders, and (relatively less frequently) IEM.

Many epileptic encephalopathy syndromes have been described based on distinctive electroclinical features that include age of onset, seizure type, and EEG pattern. For instance, Ohtahara syndrome and Early Myoclonic Encephalopathy occur in the neonatal period and early infancy, West syndrome occurs in infancy, and Lennox–Gastaut syndrome is described in older children. The electroencephalographic findings in infants with epileptic encephalopathies tend to evolve with the age of the child, based on the on the degree of brain maturation [6]. For instance, the EEG may show a burst-suppression pattern in the neonatal period, hypsarrhythmia in infancy, and evolution to multifocal or generalized slow spike-wave discharges in early childhood [7]. The clinical and electroencephalographic (EEG) features thus tend to mirror the specific age-related epileptic syndrome [8]. In other words, the distinctive electroclinical features of an epileptic syndrome are not specific to any one IEM, i.e., different IEM may share an epilepsy syndrome phenotypic features.

It is also important to remember that, in many situations, epilepsy secondary to an IEM presents with variable clinical and electroencephalographic features, making the classification as an epileptic syndrome difficult [9]. Seizure semiology can be highly variable; nevertheless, the presence of certain types of seizures (myoclonic seizures, tonic spasms) and characteristic electroencephalographic patterns (persistent burst-suppression in the absence of any medication effects) are clues that suggest IEM should be an early consideration [7].

## 4. Classification of Epilepsies Associated with Inborn Errors of Metabolism

Different approaches have been adopted in the classification of Epilepsy associated with IEM (EIEM). Assignment to a category could occur based on a specific biochemical pathway or organelle involved, e.g., disorders of amino acid metabolism, carbohydrate metabolism, urea cycle defects, lysosomal storage disorders, neurotransmitter disorders, etc. Alternatively, a pragmatic approach is suggested to classify these by age at presentation into disorders presenting in the (1) neonatal period and early infancy (2) late infancy and childhood; and (3) adolescent and adult disorders (Table 1) [10,11]. However, it must be emphasized that there can be considerable overlap in the age at presentation for many disorders while other conditions, e.g., mitochondrial disorders, could present at any age across an individual’s life span.

## 5. Clinical Presentations of Epilepsy in Inborn Errors of Metabolism

IEM can present with epileptic seizures with developmental delay preceding or following the onset of seizures in neonatal period and infancy; or in later life as neurodegenerative disorders (e.g., neuronal ceroid lipofuscinosis). Seizures may be triggered in the course of a disorder, during periods of stress, e.g., due to intercurrent illnesses, or metabolic complications such as hypoglycemia [12]. Other presentations include; dysmorphic features, changes in physical appearance and/or organomegaly (Table 2).

The clues to the presence of IEM in children with epilepsy include genetic etiologies such as a positive family history, parental consanguinity, developmental delay/regression, failure to thrive, an episodic and fluctuating course of illness, presence of other neurological abnormalities (such as movement disorders, abnormal oculomotor movements, tone abnormalities, or ataxia), change in the texture of skin, hair and nails, craniofacial dysmorphic features, ophthalmological problems (cataracts, retinitis pigmentosa, etc.), and any systemic visceromegaly (hepatosplenomegaly, cardiomegaly, etc.). Very rarely, some IEM may present as epilepsy in an otherwise normal child, e.g., early onset absence and myoclonic astatic epilepsy in glucose transporter defect.

## 6. EIEM Presenting in the Neonatal Period and Early Infancy

The occurrence of seizures is most frequent in the neonatal period as compared to other age groups, given the unique vulnerability of the brain in terms of the excitation–inhibition imbalance. While common etiologies include birth asphyxia, hypoglycemia, hypocalcemia, intraventricular hemorrhage, and meningitis, IEM may present in the neonatal period with or without encephalopathy in the form of poor feeding and lethargy, accompanied by seizures. Excessive irritability, abnormal crying, abnormal sleep, and hiccups are typical clinical markers for the clinician to suspect an underlying IEM. Clinical clues that suggest IEM being a likely etiology for the seizures and/or encephalopathy in the neonatal period are listed below [3]. We discuss selected EIEMs in detail as these manifest with epilepsy as a primary manifestation and some are potentially treatable.

Clues to the presence of IEM in the neonatal period:

- Parental consanguinity
- Deterioration after a period of apparent normalcy
- Family h/o neonatal deaths, neurological illnesses
- Pregnancy–HELLP syndrome (FAOD), excessive fetal movements (intrauterine seizures)
- Rapidly progressive encephalopathy
- Severe metabolic acidosis
- Hiccups
- Unusual odors of urine, cerumen-rare

### 6.1. Pyridoxine-Dependent Epilepsy

Pyridoxine-dependent epilepsy (PDE) is characterized by early onset recurrent seizures that are resistant to conventional anti-epileptic drugs but responsive to pyridoxine [13]. Hunt et al. first described PDE in a newborn with drug-resistant seizures which responded immediately to the administration of a multivitamin cocktail containing vitamin B6 in 1954 [14]. The underlying molecular genetic defect was identified in 2006, to be causally linked to pathogenic mutations in *ALDH7A1* gene resulting in the deficiency of α–aminoadipic semialdehyde dehydrogenase (antiquitin), which is involved in cerebral lysine metabolism [15]. Antiquitin deficiency results in the accumulation of intermediary substrates (α–aminoadipic semialdehyde (AASA), Δ-1-piperidine-6-carboxylate (P6C) and pipecolic acid) generated in the lysine degradation pathway. A condensation reaction (Knoevenagel) with P6C leads to the inactivation of pyridoxal 5′ phosphate (PLP) leading to a deficiency [16,17].

PDE usually presents very early, within hours or days of birth, with seizures that are refractory to conventional anti-epileptic (AED) therapy. In some patients, intrauterine seizures have been reported to occur, with onset at the end of the last trimester, with mothers perceiving excessive and jerky fetal movements. Infants may also develop emesis, abdominal distention, sleeplessness presenting as sepsis, or with features of hyperalertness, hyperacusis, irritability, paroxysmal facial grimacing, and abnormal eye movements [15]. Multifocal and generalized myoclonic jerks, often intermixed with tonic seizures, and focal onset motor seizures are typical initially. If left undiagnosed and untreated, or in the case of non-response to pyridoxine, affected infants develop focal dyscognitive seizures, infantile spasms, and myoclonic seizures that are treatment-resistant. Late onset and other atypical presentations of PDE have also been described in one-third of patients. These include infants with a delayed presentation (usually with infantile spasms), infants whose seizures initially respond to conventional anti-epileptic drugs, but relapse later with refractory seizures, and patients whose seizures are not controlled by initial administration of pyridoxine but respond later to a second trial [18].

Ictal and interictal EEGs in PDE pyridoxine-dependent epilepsy are variable and relatively non-specific, and may even be reported as normal [15]. However, asynchronous bursts of high-voltage generalized epileptiform activity, multifocal discharges, slow-spike wave complexes, burst-suppression pattern, and hypsarrhythmia (in infants with West syndrome presentation) have also been described [15].

PDE patients also develop neurodevelopmental disabilities, ranging from mild to severe developmental delay and intellectual disability, which usually affects the expressive language domain, associated with a low-normal motor and performance IQ scores [19]. Magnetic resonance imaging studies of the brain are variable, ranging from normal to the presence of white matter signal abnormalities, generalized cerebral atrophy, and hypoplasia or dysgenesis of corpus callosum [6,15].

Diagnostic confirmation of PDE is done through demonstration of elevated levels of AASA in urine and ⁄ or plasma and cerebrospinal fluid (CSF) [15]. Initiation of treatment with pyridoxine does not interfere with this assay. The molecular diagnosis is confirmed with the identification of a pathogenic mutation in the *ALDH7A1* gene.

Treatment should be initiated in an intensive care setting with available ventilator support as these infants may develop apnea, profound hypotonia, and hypotension in response to the administration of pyridoxine [20]. An initial dose 100 mg of pyridoxine can be given intravenously under careful monitoring. This should be followed by oral pyridoxine supplementation (30 mg/kg/day in two divided doses) for 3–7 days [11]. The duration of treatment is important before concluding the seizures are not pyridoxine-responsive as delayed responses occur. Pyridoxal phosphate (PLP) is also effective, but its use is limited by access to the product in different regions and countries and higher cost as compared to pyridoxine.

If the treatment is successful and/or the diagnosis confirmed by biochemical and/or molecular genetic testing, pyridoxine treatment must be continued indefinitely. Pyridoxine treatment has been associated with sensory peripheral neuropathy; thus, annual monitoring of nerve conduction is recommended where testing is possible [11]. If there is any evidence of abnormality on neurophysiological testing or clinical symptoms of neuropathy, the dose of pyridoxine should be reduced to the lowest effective dose. Recently, the addition of a lysine-restricted diet has been shown to be potentially beneficial, as it reduces the levels of the neurotoxic AASA [18]. An additional therapeutic option is high-dose arginine supplementation, which works by competitive inhibition of lysine uptake in the gut and the blood brain barrier [6,11].

In the long term, most treated children with PDE remain seizure-free; however, some children may have breakthrough seizures during periods of intercurrent infection and fever. In such situations, doubling the regular dose of pyridoxine during the first few days of a febrile illness may be effective at preventing breakthrough seizures [6,21].

Recently, a new genetic condition has been found to be responsible for pyridoxine-dependent epilepsy. Whole-exome sequencing of two children from a consanguineous family with pyridoxine-dependent epilepsy revealed a homozygous nonsense mutation in proline synthetase co-transcribed homolog (bacterial), *PROSC*, which encodes a PLP-binding protein of hitherto unknown function [15]. Subsequent sequencing of 29 unrelated individuals with pyridoxine-responsive epilepsy identified four additional children with biallelic *PROSC* mutations. Pre-treatment cerebrospinal fluid samples showed low PLP concentrations and evidence of reduced activity of PLP-dependent enzymes. Although the mechanism involved is not fully understood, the authors suggested that *PROSC* is involved in intracellular homeostatic regulation of PLP, supplying this cofactor to apoenzymes while minimizing any toxic side effects.

### 6.2. Pyridox(am)ine 5′-Phosphate oxidase (PNPO) Deficiency

Pyridox(am)ine 5′phosphate oxidase (PNPO) is essential for the synthesis of pyridoxal phosphate (PLP), which is the active form of vitamin B6. Deficiency of this enzyme has been described in a small number of infants worldwide who presented with seizures that were pyridoxine-resistant but PLP-responsive. Babies with PNPO deficiency are often premature, presenting with encephalopathy, seizures, lactic acidosis, and hypoglycemia [11]. The seizure semiology and EEG findings described are similar to those encountered in PDE. Maternal reports of in utero seizures are frequent. A burst-suppression pattern on EEG is frequently encountered in comparison to PDE. In contrast to PDE, breakthrough seizures while on PLP are frequently observed and patients may be sensitive to precise time intervals employed in daily PLP dosing schedule [22]. If left untreated, the disorder results in death or profound developmental impairment, with global brain atrophy and an abnormal pattern of myelination [6]. In patients identified and treated early, the outcome is usually much better [6]. Unlike PDE, PNPO deficiency lacks a specific biochemical marker in body fluids, but can be suspected on the basis of assays in blood and urine suggestive of l-aromatic acid decarboxylase deficiency (elevations in glycine, threonine, taurine, histidine, and low arginine) and treatment resistance to pyridoxine [23]. However, a definitive diagnosis can only be established by molecular genetic testing for mutations in the *PNPO* gene.

It is noted that patients carrying certain mutations in the *PNPO* gene are associated with pyridoxine responsiveness, but display resistance to treatment with PLP [6]. These patients had refractory seizures that respond to pyridoxine but had normal biomarkers and no pathogenic mutations in the antiquitin gene. They were demonstrated to have mutations in the *PNPO* gene. Two of these patients developed status epilepticus when they were switched to PLP. The authors hence recommend sequential trial of pyridoxine followed by PLP in all newborns with refractory seizures.

### 6.3. Folinic Acid-Responsive Seizures

Folinic acid-responsive seizures were first described in a group of newborns who had seizure onset in the first five days of life, along with irritability and white matter abnormalities on brain magnetic resonance imaging (MRI) [24]. These infants also demonstrated a characteristic unidentified ‘peak’ (“peak X”) on HPLC analysis of body fluids [25]. The seizures in these infants responded to folinic acid. Subsequently, other infants with similar features were identified whose seizures responded to pyridoxine. These infants were diagnosed with antiquitin deficiency, with elevated urinary AASA and pathogenic mutations in the antiquitin gene [11]. Folinic acid-responsive seizures and PDE due to antiquitin deficiency are currently considered genetically allelic disorders. The ‘peak X’ on high-performance liquid chromatograms, however, remains unidentified. The current recommendation is that, in a neonate with seizures who demonstrates an incomplete pyridoxine response, add-on treatment with folinic acid (3–5 mg/kg/day) should be considered [26].

### 6.4. Biotinidase and Holocarboxylase Synthetase Deficiency

Biotinidase is a cofactor for four carboxylases in humans. Biotinidase deficiency is an autosomal recessively inherited neurocutaneous disorder with an impairment of the endogenous recycling of biotin [11]. Epilepsy is frequent in untreated biotinidase deficiency, often starting after the first three or four months of life, as infantile spasms; optic atrophy and hearing loss are also commonly associated. Dermatological clues to the diagnosis include seborrheic dermatitis and alopecia.

Clinically, affected children present with hypotonia, seizures, eczematous skin rash, and alopecia. Some children may develop respiratory problems, such as hyperventilation, laryngeal stridor, and apnea. Conjunctivitis and candidiasis can be additional features. Later neurodevelopmental problems may occur in the form of ataxia, developmental delay, hearing loss, and vision problems such as optic atrophy [18]. Biochemical abnormalities may include one or more of the following; ketosis, lactic acidosis, organic aciduria, and mild hyperammonemia. The seizures and skin manifestations respond promptly to small doses of biotin, usually 5–20 mg/day. The neurodevelopmental sequelae such as developmental delay, optic atrophy, and hearing loss are usually irreversible. A few reports in untreated children include presentations with myelopathy and progressive spastic paresis. In holocarboxylase synthase deficiency, symptoms start during the neonatal period. Seizures are less frequent, occurring in 25–50% of all children [27]. Biotin is effective, although a higher dose may be needed in some cases. Many newborn screening programs include biotinidase deficiency because of the availability of a robust and inexpensive screening test and the ease and efficacy of treatment with biotin.

### 6.5. Glucose Transporter Defect (GLUT1 Deficiency Syndrome)

The deficiency of cerebral glucose transporter (GLUT1) leads to the GLUT1 deficiency syndrome, resulting in impaired glucose transport into the brain. Glut-1 deficiency is generally caused by autosomal dominantly inherited or sporadic haploinsufficiency of the blood/brain barrier passive glucose transporter, Glut-1 (*SLC2A1* gene) but cases of autosomal recessive inheritance have been described [28].

Classically, GLUT1 deficiency syndrome presents with infantile onset refractory seizures, developmental delay, acquired microcephaly, abnormalities of muscle tone (hypotonia or spasticity), and movement disorders such as choreoathetosis, ataxia, and dystonia [12]. The clinical spectrum of GLUT1 deficiency is understood to be broader and includes developmental delay, epilepsy, and familial and sporadic forms of paroxysmal exercise-induced dyskinesia [6]. Varying degrees of cognitive impairment associated with dysarthria, dysfluency, and expressive language deficits are added features [28].

Patients usually appear normal at birth, and present with refractory focal motor seizures in early infancy. In addition, especially before meals or at times of fasting, patients may exhibit an episodic movement disorder with associated eye-rolling movements [28]. About 60% of infants develop acquired microcephaly [28]. Beyond infancy, generalized seizures are common. The EEG may be completely normal; recordings after an overnight fast can be used as an activation procedure to demonstrate the altered background activity or focal discharges [6].

Two types of generalized epilepsy syndromes have been reported to be seen in GLUT1 deficiency early onset absence epilepsy in young children and myoclonic astatic epilepsy [6]. Lebon et al. reported the results of GLUT1 deficiency genetic testing in patients with idiopathic generalized epilepsy and pooled their data with other similar studies [6]. Of 1110 screened patients with generalized epilepsies from seven studies, 2.4% were found to have GLUT1 deficiency, while 5.6% of 303 patients with early onset absence epilepsy from four studies had GLUT1 deficiency. Additional features described in GLUT1 deficiency patients include abnormal movements (50%), a family history of seizures (41%), or both. GLUT1 deficiency was more likely to be found in patients with myoclonic astatic epilepsy and early onset absence epilepsy. The authors concluded that the probability of finding GLUT1 deficiency in the classical idiopathic generalized epilepsies is very low.

Clinical clues to GLUT1 deficiency include an increase in seizures before meals, cognitive impairment, or paroxysmal exercise-induced dyskinesia [29]. In rare cases, focal epilepsy has also been reported in GLUT1 deficiency syndrome [30]. The clinical pointers in such cases are the presence of cognitive impairment and/or paroxysmal exercise-induced dyskinesia.

The diagnosis is suggested by the finding of low CSF glucose levels in the absence of other causes of hypoglycorrhachia such as infection. Earlier CSF to blood glucose ratios <0.4 were considered diagnostic; however, the use of absolute CSF sugar values below 2.22 (to 3.33) mmoles/L is considered to be more sensitive [30]. Lumbar puncture should be performed in the fasting state. Blood or plasma glucose should be drawn immediately prior to the lumbar puncture in order to minimize stress-related hyperglycemia [31]. As many GLUT1 deficiency patients with normal CSF sugar levels have been described, molecular genetic testing for pathogenic mutations in the *SLC2A1* gene may be considered the gold standard and molecular diagnostic testing should be considered whenever there is a strong suspicion on clinical grounds.

The ketogenic diet is an effective treatment for this condition as it provides alternative sources of energy substrate in the form of ketone bodies that the brain preferentially utilizes [3]. The fat to non-fat ratio in the diet is usually 3:1 to 4:1. The modified Atkins diet, which is a less restrictive alternative, may be considered in children or adolescents with a milder phenotype. Drugs that impair GLUT1 function, e.g., caffeine, phenobarbital, diazepam, chloral hydrate, and tricyclic antidepressants, are best avoided [11]. Recently, triheptanoin, an anaplerotic agent that replenishes the Krebs cycle metabolites, has been found useful in reducing the non-epileptic paroxysmal manifestations and normalizing the brain bioenergetics profile in patients with GLUT1 deficiency syndrome [32].

### 6.6. Serine Biosynthesis Disorders

Three enzymes are involved in serine biosynthesis, 3-phosphoglycerate dehydrogenase (3-PGDH), 3-phosphoserine aminotransferase (PSAT), and phosphoserine phosphatase (PSP) [28]. Common clinical features of defects in serine biosynthesis include congenital or early acquired microcephaly, refractory seizures and global developmental delay in infancy, and progressive polyneuropathy in adults [33]. Seizure types include; infantile spasms (in one-third), tonic–clonic seizures, tonic seizures, atonic seizures, gelastic seizures, and myoclonic seizures. Other clinical features variably present include cataracts, spastic quadriparesis, megaloblastic anemia, and irritability [34]. The brain MRI may show delayed myelination followed by cerebral atrophy.

Low CSF serine levels are seen in all three disorders. Plasma serine levels may be low to borderline. The diagnosis can be confirmed by molecular genetic testing. Treatment consists of supplementation with serine (up to 700 mg/kg/day) and glycine (up to 300 mg/kg/day) [35]. Seizures may respond to serine supplementation. Normal neurodevelopmental outcomes have been reported in prenatally treated patients with 3-PGDH and PSAT deficiency [35].

Neu–Laxova syndrome has recently been shown to be a serine biosynthesis defect caused by PHGDH or PSAT1 deficiency [34]. This syndrome is characterized by dysmorphic facies, microcephaly, intrauterine growth restriction, skin abnormalities (ichthyosis and hyperkeratosis), and flexion deformities of the limbs.

### 6.7. Molybdenum Cofactor and Isolated Sulfite Oxidase Deficiency

Both these disorders share similar clinical features characterized by the onset of intractable seizures, encephalopathy, and hypotonia in the neonatal period, often mimicking neonatal hypoxic ischemic encephalopathy. Subtle dysmorphic features such as elongated facies, small nose, and puffy cheeks may be present [34]. Later, the affected infants develop epileptic seizures, developmental delay, and movement disorders. Clinical clues to this disorder in infancy include ophthalmological abnormalities such as lens subluxation, optic atrophy, or nystagmus [36]. EEGs may show multifocal spike wave discharges or a burst suppression pattern. The MRI shows generalized brain edema in the early in the early stage, and a distinctive pattern of widespread restricted diffusion involving the cortical ribbon (at the depths of sulci), followed by development of cystic changes of the white matter and global brain atrophy [6]. The MRI pattern of multicystic encephalomalacia resembles that seen in perinatal hypoxic ischemic brain injury. Biochemical investigations confirm the low levels of uric acid and homocysteine in plasma and elevated Urinary S-sulfocysteine [6].

In two-thirds of patients with molybdenum cofactor deficiency, a proximal defect in the pathway of molybdenum cofactor synthesis is responsible, leading to the failure of conversion of guanine triphosphate (GTP) to cyclic pyranopterin monophosphate [37]. A new treatment is available for these patients using purified intravenous cyclic pyranopterin monophosphate (cPMP). Reduction in seizures frequency and severity and improved developmental outcome, along with correction of biochemical abnormalities has been demonstrated [38]. However, this treatment appears to be helpful when started early in life, before permanent brain damage has set in. In the remaining one-third patients who have a distal defect, and for patients with sulfite oxidase deficiency, no definitive treatment is currently available [11].

### 6.8. Menkes Disease

Menkes disease is an X-linked recessive disorder of copper metabolism. Clinical clues to Menkes disease include hypotonia, seizures, cutis laxa, “kinky hair,” and hypothermia. The characteristic hair abnormality ‘pili torti’ on microscopic examination of hair is a diagnostic clue. Widespread cerebral and cerebellar degeneration, tortuosity of blood vessels can be demonstrated on neuroimaging (MRI, MRA), as well as bladder diverticulae, and skeletal abnormalities (presence of wormian bones on skull X-rays) [39].

Menkes disease is caused by mutations in the *ATP7A* gene, a ubiquitous copper transporter encoded on the X-chromosome and located in the trans-Golgi network, particularly active in the intestine, from which most of the dietary copper is absorbed. The impairment of copper transport leads an intracellular copper deficit, resulting in the dysfunction of copper-dependent enzymes, e.g., mitochondrial cytochrome c oxidase, lysyl oxidase, superoxide dismutase, dopamine-β-hydroxylase, and tyrosinase. Secondary elevations in plasma lactate are often noted and related to cytochrome c oxidase deficiency [11]. Characteristic hair abnormalities such as pili torti and trichorrhexis nodosa occur as a result of altered molecular bridges of collagen (lysyl oxidase). Other features include abnormalities in the intracranial vasculature (elongation and tortuosity of intracranial vessels and subdural haematoma), and Purkinje cell degeneration in the cerebellum [40,41].

The disease is characterized by the appearance of seizures and developmental arrest and regression often around 6–8 weeks of age [40]. Three distinct stages appear to characterize the evolution of epilepsy in Menkes disease [6,41]. The first stage (at 2–3 months of age) consists of focal seizures that evolve into status epilepticus. Typical EEG changes include focal epileptiform and ictal rhythms over posterior quadrants. Interictal abnormalities include polymorphic slow, spike and waves and multifocal epileptiform discharges. Three to eight months later, epileptic spasms occur with EEG changes consistent with hypsarrhythmia. Finally, a third late stage occurs (at 20–25 months of age) characterized by the development of multifocal seizures, tonic spasms, and myoclonus [42]. The late stage EEG shows multifocal polymorphic activity with intermixed irregular slow waves.

The diagnosis is suggested by low serum levels of copper and ceruloplasmin and the presence of neuroimaging abnormalities described above, and confirmed by molecular genetic testing for pathogenic mutations in the *ATP7A* gene [41].

Therapy with parenteral copper histidine has prolonged life expectancy if started early, but does not appear to significantly alter the neurological outcome. Copper supplementation may be more effective in cases where the causative mutation leads to a protein that retains some residual function [40].

### 6.9. Nonketotic Hyperglycinemia

Nonketotic hyperglycinemia is caused by deficiency of the glycine cleavage enzyme system and consequent accumulation of glycine in the brain. The classic form of nonketotic hyperglycinemia present in the newborn period with hypotonia, feeding difficulties, encephalopathy, seizures, and apneas [43]. The presence of hiccups is an important clinical clue [41]. Characteristically EEG recordings show a burst suppression pattern which later evolves to hypsarrhythmia and multifocal epilepsy [44]. On magnetic resonance imaging (MRI), with a classic newborn presentation, infants show increased signal on diffusion weighted images in the areas that are myelinated at birth, most often in the posterior limb of the internal capsule [45]. Hypoplasia or agenesis of the corpus callosum has also been reported. Magnetic resonance spectroscopy demonstrates a glycine peak in the proton MRS spectra.

One-third of patients have a delayed presentation; in early to mid-infancy with seizures, hypotonia, and developmental delay [46]. In the long term, the majority of patients have a poor outcome with severe global developmental delay and spasticity. They also develop progressive brain atrophy and multifocal seizures over time. The diagnosis is performed by measuring glycine concentrations in plasma and CSF. There is an increased ratio of CSF to plasma glycine >0.04 [47]. The definitive diagnosis again is established by molecular genetic testing. Therapy with sodium benzoate and dextromethorphan (NMDA receptor antagonist) may be helpful in some milder forms of the disease, alongside AED and general supportive care [44]. Sodium valproate must not be used as it further inhibits glycine metabolism [6].

### 6.10. Uridine-Responsive Epileptic Encephalopathy

This is a newly described disorder of pyrimidine biosynthesis caused by CAD mutations [11]. CAD encodes a multifunctional enzyme (CPSase/ATCase/DHOase) involved in de novo pyrimidine biosynthesis. Pyrimidines can also be recycled from uridine. Exome sequencing in three families identified biallelic CAD mutations in four children with global developmental delay, epileptic encephalopathy, and anemia with anisopoikilocytosis [6]. Two died aged four and five years after a neurodegenerative disease course. Supplementation of the two surviving children with oral uridine led to immediate cessation of seizures in both. A dramatic improvement was also noted in two children who were previously in a minimally conscious state and began to communicate and walk with assistance after nine weeks of treatment. There was also normalization of the peripheral blood smears and resolution of anemia.

### 6.11. Urea Cycle Defects, Organic Acidemias, and Aminoacidopathies

These disorders present either in the neonatal period with encephalopathy and seizures, or later episodes of encephalopathy, neurological worsening (ataxia, movement disorders, etc.) during periods of stress such as with intercurrent infections.

Urea cycle disorders represent disorders in the metabolic pathway of ammonia detoxification into urea. The enzymes in the urea cycle pathway include: carbamoyl phosphate synthase I (CPSI); ornithine transcarbamylase (OTC); argininosuccinic acid synthetase (ASS); argininosuccinic acid lyase (ASL); arginase (ARG); and the allosteric activator of CPSI, *N*-acetyl glutamate synthetase (NAGS). OTC deficiency is inherited in an X-linked recessive mode of inheritance, with an autosomal recessive inheritance pattern in the remainder [48].

Babies with urea cycle defects are normal at birth but soon develop lethargy, poor feeding, seizures, and tone abnormalities in the first 4–7 days of life. The EEG shows variable and nonspecific patterns of multifocal independent spike- and sharp-waves, repetitive paroxysmal discharges and low-voltage fast activity [48]. Diagnosis is suspected by the presence of high ammonia levels in the absence of metabolic acidosis or ketosis and abnormal plasma concentrations of urea cycle intermediates and urine orotic acid. Diagnostic confirmation is by testing for the deficient enzyme activity and molecular genetic testing.

In milder variants with partial deficiency of the enzyme, the affected children may have intermittent episodes of encephalopathy, ataxia, and/or seizures during periods of metabolic stress and concurrent infections. In argininosuccinic aciduria, a more chronic presentation with trichorrhexis nodosa, irritability, rigidity, and refractory seizures is common [12].

Organic acidemias such as methylmalonic aciduria, propionic aciduria, etc. may present either in the neonatal period or beyond the neonatal period with a presentation similar to urea cycle defects. The biochemical hallmark is the presence of significant metabolic acidosis with or without ketosis and hyperammonemia. Basal ganglia signal abnormalities may be seen on the MRI. The diagnosis is established by means of quantitative analysis of plasma amino acids and acylcarnitines, as well as urinary organic acids [12]. Early diagnosis and specific treatment (special dietary formulae, cofactor supplementation) are essential to improve seizure management and prevent irreversible long-term sequelae.

Disorders that present with features like methylmalonic aciduria include intracellular defects of cobalamin C/D (Vitamin B12) metabolism. These disorders are characterized by the functional deficiency of methylmalonyl CoA mutase and methionine synthase. As cobalamin is a co-factor for these enzymes, the functional deficiency leads to excretion of methylmalonic acid and homocysteine in the urine, respectively. The clinical features include seizures, hypotonia, developmental delay, microcephaly, megaloblastic anemia, and, rarely, hemolytic uremic syndrome [49,50]. Treatment with hydroxycobalamin, betaine and carnitine may be beneficial.

The classic variant of maple syrup urine disease also presents in the neonatal period with encephalopathy, tone abnormalities, and/or seizures, usually at the end of the first week of life. There is an absence of acidosis or hyperammonemia; the biochemical diagnostic clue is the presence of urine ketones. The MRI shows localized edema involving the myelinated parts of the brain such as the deep cerebellar white matter, the dorsal part of the brainstem, the cerebral peduncles, and the posterior limb of the internal capsule [6]. The EEG pattern is variable and it includes spikes, polyspikes, spike-wave complexes, triphasic waves, comb-like rhythm, severe slowing, and bursts of periodic suppression [6].

### 6.12. Congenital Disorders of Glycosylation

Congenital disorders of glycosylation (CDG) are multisystem disorders caused by genetic defects in glycoprotein, glycolipid, and glycan synthesis. Neurological features are common and include developmental delay, seizures, stroke-like episodes, hypotonia, ataxia, and peripheral neuropathy in CDG1A, the type most frequently encountered [51]. Seizures have been reported in almost all types of CDG. The clinical pointers to an underlying CDG include dysmorphic features such as unusual fat pads on the buttocks, inverted nipples, long fingers and toes, and craniofacial dysmorphic features [52].

In a series of 27 patients with CDG, myoclonic, clonic, and focal seizures were noted at presentation [53]. Over time, these individuals developed recurrent and intractable epilepsy with heterogeneous seizure semiology including; tonic–clonic seizures, infantile spasms, and myoclonic seizures. The EEG abnormalities described in these patients included focal and multifocal epileptic discharges, slow background rhythms, generalized epileptic activity, and burst suppression pattern [11]. The diagnosis of many CDGs is established by isoelectric focusing of serum transferrin. Treatment interventions are symptomatic as no specific treatment measures are currently available.

### 6.13. Peroxisomal Disorders

Peroxisomes are intracellular organelles that serve as functional sites for several metabolic pathways, namely plasmalogen biosynthesis and oxidation of branched-chain and very-long-chain fatty acids (VLCFAs) [53]. Peroxisomal disorders are multisystem disorders and those associated with early onset seizures include Zellweger syndrome, neonatal adrenoleukodystrophy, and infantile Refsum’s disease. The pathogenesis of seizures in these patients includes neuronal migration defects, biochemical abnormalities such as aberrant fatty acid composition of neuronal membranes, and dysregulation of GABAergic signaling [53]. Craniofacial abnormalities, eye abnormalities, neuronal migration defects, hepatomegaly, and chondrodysplasia punctate are features of peroxisomal disorders. Seizures occur in 70–90% of the patients and are difficult to control [54]. Diagnosis is established by demonstration of elevated plasma VLCFA levels and relevant pathogenic mutation in one of the Peroxisomal Biogenetic Factor (*PEX*) genes. Treatment remains supportive.

### 6.14. Congenital Disorders of Autophagy

These conditions constitute a new subclass of IEM that affect the autophagy pathway. Autophagy is the umbrella term for essential intracellular pathways that deliver cytosolic cargo to lysosomes for degradation [54]. Clinically, these disorders affect the brain, leading to intracranial malformations, developmental delay, intellectual disability, epilepsy, movement disorders, and neurodegeneration [55]. Disorders of autophagy include Vici syndrome, β-propeller protein-associated neurodegeneration due to mutations in *WDR45* (WD repeat domain 45) gene, *SNX14* (Sorting Nexin 14)-associated autosomal recessive cerebellar ataxia and intellectual disability syndrome, and variants of hereditary spastic paraplegia. Diagnosis can only be established by timely recognition of the clinical phenotype and molecular genetic testing for mutations in the affected gene.

## 7. Epilepsies Associated with Inborn Errors of Metabolism Presenting in Late Infancy and Childhood

In this age group, the disorders mentioned in the previous section may present as late-onset variants with developmental delay and epilepsy; clinical clues are summarized below. These features may overlap with additional presentations that include movement disorders, behavioral problems, neuropsychiatric symptoms, and symptoms suggestive of autistic spectrum disorder. Finally, grey matter neurodegenerative disorders such as GM1 gangliosidosis and neuronal ceroid lipofuscinosis, particularly CLN1 (neuronal ceroid lipofuscinosis type 1) and CLN3 (neuronal ceroid lipofuscinosis type 3) forms, also present neurodevelopmental regression and development of seizures during the progression. A few other disorders are worthy of consideration.

Clues to an underlying IEM in epilepsies presenting in adolescence/adulthood [56]:

- Positive family history
- Progressive myoclonic epilepsy phenotype
- Epilepsy not fitting into a classical epilepsy syndrome
- Associated intellectual disability/ cognitive decline
- Presence of other neurological features: movement disorders, ataxia, spasticity
- Presence of other system involvement: hepatosplenomegaly, retinitis pigmentosa
- Seizures worsen with certain anti-epileptic drugs
- Unexplained status epilepticus
- Seizures occurring on fasting (GLUT1) or with high protein meals (urea cycle defects)
- Unexplained slowing of the background activity on EEG
- Paroxysmal responses during the photic intermittent stimulation at low frequencies on EEG

### 7.1. Disorders of Creatine Biosynthesis and Transport

Cerebral creatine deficiency states comprise two autosomal recessive disorders that affect creatine biosynthesis: arginine:glycine amidinotransferase (AGAT) deficiency and guanidinoacetate methyltransferase (GAMT) deficiency, and creatine transporter (SLC6A8) deficiency, an X-linked condition that affects creatine transport into brain and muscle [57]. The shared clinical features amongst these disorders include; developmental delay/intellectual disability, behavior problems, autistic features, speech delay, epilepsy, and movement disorders [57]. Amongst these disorders, seizures are most frequent in GAMT deficiency, occurring in over 90% of affected patients [58].

Multiple seizure types have been described in these children including myoclonic, generalized tonic–clonic, focal onset with impaired awareness, head nodding, and drop attacks. Neurological findings include hypotonia in early stages, with dystonia in the later stage. EEG may show high amplitude slow background with multifocal spikes [58].

The MRI of the brain shows an abnormal hyperintense signal in the globi pallidi on T2 weighted sequences. A marked reduction of the creatine signal peak on proton magnetic resonance spectroscopy (MRS) is demonstrable. Urinary guanidinoacetate (GAA) levels are elevated. Intracellular accumulation of GAA in the brain is considered to be both neurotoxic and epileptogenic [11].

Early recognition and diagnosis is important, as this is a treatable disorder. The goal of treatment in GAMT deficiency is to increase creatine levels and to reduce GAA accumulation [58]. Oral supplementation of creatine monohydrate (400 mg/day) is used to replenish deficient cerebral creatine levels. Therapeutic strategies to reduce GAA levels include high-dose l-ornithine supplementation to competitively inhibit AGAT activity, and dietary arginine restriction to induce substrate deprivation [59]. Sodium benzoate may also be beneficial as it reduces the production of GAA via conjugation with glycine to form hippuric acid, which is subsequently excreted by the kidneys [6].

### 7.2. Adenylosuccinate Lyase (ADSL) Deficiency

ADSL deficiency is an autosomal recessive disorder of purine metabolism. Adenylosuccinate lyase deficiency type I is most common, and is characterized by severe psychomotor retardation, early onset seizures and microcephaly [58]. Seizure semiology is variable including myoclonus, focal onset seizures with or without impaired awareness, epileptic spasms, and status epilepticus. Concurrent autistic features are frequently seen. MRI may show non-specific abnormalities such as cerebral atrophy and delayed myelination [58]. Demonstration of the abnormal purine metabolites in the urine and molecular genetic testing for pathogenic mutations in the *ADSL* gene can provide diagnostic and genetic confirmation. Therapeutic interventions that may be of value include d-ribose administration, which increases the provision of phosporibosylpyrophosphate (PRPP), which in turn increases de novo purine synthesis [60].

### 7.3. Lysosomal Storage Disorders

Lysosomal storage disorders presenting in this age group with seizures include conditions such as neuronal ceroid lipofuscinosis (NCL), gangliosidosis (GM1, GM2), Niemann–Pick disease, Gaucher’s disease and sialidosis. The early features are of a child with cognitive decline and seizures, while motor regression occurs later. The neuronal ceroid-lipofuscinoses are characterized by progressive intellectual deterioration, seizures, and vision impairment without organomegaly. NCL variants are classified by age of onset as congenital, early infantile, late infantile, juvenile, adult, and variant NCL [61]. The various clinical features of some of the common variants are summarized in Table 3.

Gangliosidosis, Niemann–Pick disease, Gaucher’s disease and sialidosis are characterized by the presence of hepatosplenomegaly in addition to seizures and cognitive decline. Coarse facies are noted in GM1 gangliosidosis. Biochemical assays of lysosomal acid hydrolases in plasma, white blood cell pellets, or cultured skin fibroblasts can establish diagnoses of many lysosomal storage diseases and the identification of pathogenic mutations by molecular genetic testing is advisable to provide genotype–phenotype correlation and counseling.

### 7.4. Mitochondrial Disorders

Epilepsy is a frequent manifestation of mitochondrial diseases, with seizures reported to occur in 35–60% of individuals with biochemically confirmed mitochondrial disease [60,62,63]. In one series of 48 patients with epilepsy and confirmed mitochondrial respiratory chain defects, the following were the epilepsy syndromic diagnoses; Ohtahara syndrome (2), West syndrome (10), Lennox–Gastaut syndrome (12), Landau–Kleffner syndrome (2), generalized epilepsy (14), and focal epilepsy (8) [64]. The epilepsy associated with mitochondrial disorders has been classified into six groups based on the age at onset and major seizure types (Table 4) [65].

In most patients with mitochondrial disorders, seizures are often preceded by or associated with other symptoms such as developmental delay, failure to thrive, ataxia, vision impairment, deafness, and evidence of multiorgan involvement. The MRI may show symmetrical signal abnormalities in the basal ganglia and/or cerebellum, cerebral and/or cerebellar atrophy, and white matter signal abnormalities. MRS studies often demonstrate an elevated lactate peak in affected regions of the brain. Explosive onset of focal epilepsy or epilepsia partialis continua in early life should raise suspicion of Alpers syndrome, particularly in cases with coexisting or later onset liver dysfunction [67]. A clue on the EEG, though non-specific, is the presence of relative predominance of occipital epileptiform discharges. A recently described EEG finding in patients with Alpers disease is the presence of rhythmic high-amplitude delta with superimposed (poly)spikes (RHADS) [66].

This diagnosis is confirmed by molecular genetic testing for POLG1 (Polymerase γ1) mutations. Seizures associated with nephritic syndrome, or renal tubulopathies and deafness may also be seen in CoQ 10 deficiency [65,68].

In recognizable phenotypes such as Alpers syndrome and MELAS, the diagnosis can be readily confirmed by mutation testing. In other suspected mitochondrial epilepsies, diagnosis requires testing of blood and CSF lactate (which may be variably elevated), MRI and MRS of the brain, muscle biopsy (histology may show ragged red fibers and cytochrome oxidase (COX) negative fibers) and analysis of the respiratory chain enzymes on the muscle biopsy [65]. Further genetic testing may be guided by the results of the respiratory chain enzyme testing and muscle histochemistry. Targeted gene panels are increasingly available in diagnostic laboratories that include genes in both the nuclear and mitochondrial genomes in a search for pathogenic mutations.

Treatment is generally supportive and symptomatic. It is important to recognize CoQ 10 deficiency as this is treatable with CoQ supplementation. The use of sodium valproate must be avoided in all patients with suspected mitochondrial epilepsies, as this carries the risk of inducing fatal hepatotoxicity [69]. Some patients may benefit from supplementation of thiamine, riboflavin, l-carnitine or coenzyme Q10. Arginine may be beneficial in reducing the stroke-like episodes in patients with MELAS [65]. Levetiracetam is the drug of choice for myoclonic seizures in MERRF syndrome and lamotrigine may have a neuroprotective effect [6,70,71]. A ketogenic diet may be beneficial in patients with refractory epilepsy associated with respiratory chain defects [72].

## 8. Epilepsies Associated with IEM Presenting in Adolescence and Adulthood

Epilepsies associated with IEM presenting in this age group can be divided into two major groups: progressive myoclonic epilepsies and other epilepsy types [65]. The second group may be further divided into two subgroups: epilepsy with and without intellectual disability/cognitive decline. There are certain clues that suggest an underlying IEM (Box 2) [73].

Progressive myoclonic epilepsy (PME) syndromes represent a distinctive electroclinical phenotype consisting of myoclonus, epileptic seizures (most often generalized tonic–clonic, but sometimes focal onset, and atypical absences), cognitive decline, cerebellar ataxia, and progressively worsening clinical course [56]. Vision impairment may be associated in many cases. The EEG background is slow, and interictal generalized epileptiform discharges are often associated with a photoparoxysmal response.

The onset of PME may mimic that of Juvenile myoclonic epilepsy (JME). PME can be caused by IEM as well as genetic degenerative brain diseases such as Unverricht–Lundborg disease and juvenile Huntington’s chorea. Several inborn errors of metabolism can cause PME including ceroid lipofuscinosis, sialidosis type 1, Gaucher disease type III, mitochondrial cytopathies, and Lafora disease [56]. Certain drugs may worsen seizures, such as phenytoin, carbamazepine, gabapentin, vigabatrin, tiagabine, and lamotrigine. Preferred drugs are benzodiazepines, valproate, levetiracetam, topiramate, zonisamide, and phenobarbital.

In the second group, epilepsy associated with intellectual disability can be caused by late-onset presentations of disorders of creatine metabolism, mitochondrial disorders, GLUT-1 deficiency, urea cycle defects, organic acidemias, succinic semi-aldehyde dehydrogenase deficiency (SSADH), and lysosomal storage disorders such as juvenile Niemann–Pick disease Type C [74]. Clues to an underlying IEM include episodes of encephalopathy (especially during periods of stress and intercurrent infections, vomiting, ataxia, movement disorders, multisystem involvement, laboratory evidence of liver disease, visceromegaly, and vision and hearing impairment.

Epilepsy without intellectual disability may be seen in Wilson’s disease and porphyria [56]. Seizures have been reported in 8.3% of patients in a case series of 490 patients with Wilson’s disease [56]. The age at onset of seizures in this series was 12.8 ± 5.7 years. Seizures preceded the onset of characteristic extrapyramidal features of Wilson’s disease in 19.5%, occurred concurrently in 46.3%, followed chelation therapy in 29.2%, or occurred as a terminal event in 4.8%. Wilson’s disease is important to recognize, as it is potentially a treatable disorder.

Acute intermittent porphyria (AIP) patients may develop seizures during the acute attacks in 5–25% of the cases [56,75]. These are usually associated with other classic AIP symptoms such as abdominal pain, psychiatric features, peripheral neuropathy, and red urine. Seizures are exacerbated by antiepileptic drugs including valproate, lamotrigine, carbamazepine, phenytoin, or topiramate and should therefore be avoided. Porphyria should also be considered if the addition of a new anti-epileptic medication causes a major deterioration in a patient’s epilepsy [76].

## 9. Approach to Diagnosis of IEM-Associated Epilepsy

IEMs are a rare cause of epilepsy, accounting for about 5–7% in retrospective studies [77]; however, they must be considered in the clinical scenarios described earlier. Commonly these include concomitant developmental delay/regression, difficult-to-treat epilepsy, the presence of other neurological abnormalities such as movement disorders and ataxia, systemic involvement, parental consanguinity, and a positive family history of a similar illness.

The evaluation should document details of the prenatal, perinatal, and postnatal periods during pregnancy. History of excessive and jerky fetal movements could suggest in utero seizures, which have been reported in pyridoxine dependency and PNPO deficiency [77]. A history of prematurity is seen in association with pyridoxal phosphate responsive seizures [2]. The age of onset of symptoms is significant; in urea cycle defects, organic acidemias, and aminoacidopathies such as MSUD, the infant is usually well at birth and symptoms present 4–7 days later by the time toxic intermediary metabolites likely accumulate. A detailed family history and a three-generation pedigree with relevant information should be assembled to assess suggestions of a Mendelian inheritance patterns.

A history of onset of illness with complex febrile seizures in infancy is characteristic of patients with Dravet syndrome; a similar presentation has also been reported with type II hyperprolinemia and Menke’s disease. Epilepsia partialis continua (especially if occurring on and off bilaterally) in a young child must lead to the diagnostic consideration of Alpers disease and other mitochondriopathies. The association of the occurrence of seizures activity with food intake/fasting (e.g. GLUT1 deficiency) must be noted.

The developmental history is important in elucidating the developmental trajectory (delay, plateau) prior to the onset of seizures and recognition of developmental arrest or regression after the onset of seizures is equally significant. Autistic traits are often reported in cerebral creatine deficiency disorders and ADSL deficiency. Systematic screening for hearing, vision abnormalities, and symptoms related to gait and movement abnormalities should be elicited.

A general physical examination to look for dysmorphic features that may point towards conditions such as peroxisomal disorders, or congenital disorders of glycosylation). Skin and hair abnormalities (Menke’s disease, biotinidase deficiency), if identified, often serve as diagnostic clues. The detection of severe anemia with (anisopoikilocytosis on blood smears) is a clue towards an underlying uridine responsive epileptic encephalopathy. A complete ophthalmological evaluation is essential to identify features of pigmentary retinopathy (NCL, mitochondrial disorders), macular cherry red spots (gangliosidosis, Niemann–Pick disease), lens dislocation (sulfite oxidase deficiency), and cataracts (serine biosynthesis defects). Systemic examination to look for organomegaly (storage disorders) must be done.

Neurological examination is essential to detect abnormalities of muscle tone, and any abnormal movements (chorea, athetosis, dyskinesias) The presence of intermittent dystonia and exercise-induced dyskinesia raise the possibility of GLUT1 deficiency syndrome. Movement disorders are also seen in association with organic acidemias, mitochondriopathies, and lysosomal storage disorders. Head circumference must be measured. Macrocephaly may be noted in glutaric aciduria and Menkes disease, while microcephaly may be noted in many IEM such as phenylketonuria, NCL, serine biosynthesis defects, and Alpers syndrome.

## 10. Investigations

**Figure 1:**
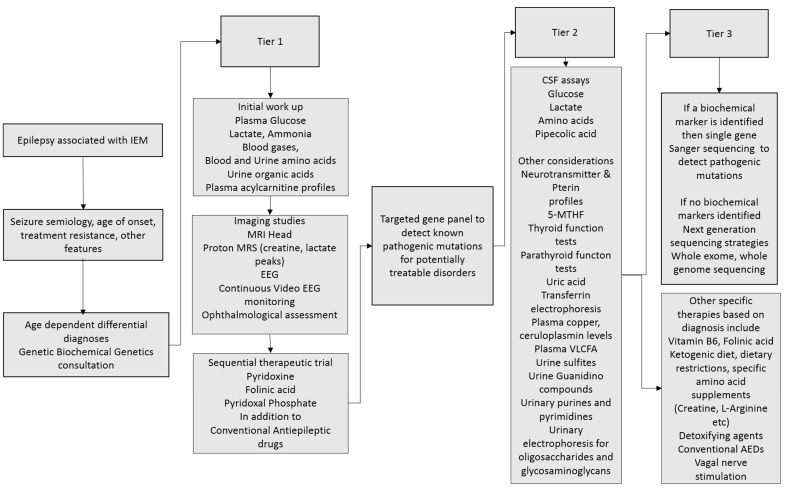
Epilepsy associated with IEM; suggested tiered approach to investigation, diagnosis, and management.

The investigative workup of a child with a suspected IEM with epilepsy is a combination of biochemical, neuroimaging, EEG, tissue biopsies (e.g., muscle biopsies in suspected mitochondrial disorders), and molecular genetic testing. A tiered approach is proposed and summarized in Figure 1.

### 10.1. Biochemical Investigations

The initial metabolic screen in all cases includes point of care testing for blood glucose, as well as electrolytes (calcium, magnesium), lactate, arterial blood gas, ammonia, and urinary ketones [78].

Biochemical screening should include assays for plasma carnitine and acylcarnitine, amino acids, and urinary organic acid profile. Abnormalities detected are helpful in the diagnosis of organic acidurias, aminoacidopathies, urea cycle defects, and primary disorders of energy metabolism (Table 5) [1,9]. Urinary AASA and CSF pipecolic acid assays are useful markers for patients with suspected pyridoxine dependency.

If the initial metabolic investigations are non-contributory, the focus of investigation and management should be directed towards the identification of potentially treatable IEM. Serum Total homocysteine levels (MTHFR deficiency) and biotinidase assays should be performed as these disorders may be missed in the metabolic screen [1]. Performing a lumbar puncture and obtaining a CSF sample is an important step in situations where other body fluid assays are negative, as it provides a window into brain neurochemistry.

CSF assays should be performed for amino acids, lactate, pipecolic acid, and 5-methyltetrahydrofolate, as well as neurotransmitter and pterin profiles [11]. Glucose and amino acids must be determined in blood and CSF simultaneously, with blood samples preferably drawn immediately prior to lumbar puncture. Cerebrospinal fluid abnormalities may point towards several inherited metabolic disorders (Table 5): low serine levels (serine biosynthesis defects), hypoglycorrhachia (glucose transporter GLUT1 deficiency), elevated lactate (mitochondrial disorders), elevated pipecolic acid, reduced 5-methylenetetrahydrofolate (cerebral folate deficiency), and abnormal pterin profile (tetrahydrobiopterin metabolism defects, pyridoxine-dependent epilepsy and peroxisomal diseases). CSF neurotransmitter assays have to be interpreted in relationship to the clinical and other biochemical assays on body fluids to identify defects in neurotransmitter biosynthesis.

### 10.2. EEG

EEG helps to clarify the seizure type and the type of epileptic encephalopathy. A video-EEG is preferable over a routine EEG to better understand and establish seizure semiology and ictal EEG patterns. Photic stimulation may demonstrate abnormal photoparoxysmal responses in infantile neuronal ceroid lipofuscinosis and progressive myoclonic epilepsies. The abnormalities seen in EEG are not specific but some abnormalities such as burst suppression may suggest of an underlying IEM. A comb-like rhythm has been described in MSUD [1]. The presence of rhythmic high-amplitude delta with superimposed (poly)spikes (RHADS) in a child with status epilepticus is a pointer towards the presence of Alpers disease [1].

### 10.3. Neuroimaging Studies

MRI of the brain must be performed in all children with epilepsy and suspected IEM. The features to be noted while evaluating an MRI for suspected IEM include signal abnormalities in the cortex, basal ganglia, brainstem, dentate nucleus, any diffusion restriction, white matter signal abnormality and its pattern, and presence of any cerebral malformations [79].

MRI may show symmetrical basal ganglia signal abnormalities in organic acidemias and mitochondriopathies. A relatively specific (and hence diagnostic) MRI pattern may be noted in some IEM such as glutaric aciduria, l-2 hydroxyglutaric aciduria, and MSUD [68]. In many IEM, the MRI may be normal or may show non-specific features such as cerebral atrophy or delayed myelination. A proton MR spectroscopy performed simultaneously is of diagnostic value in many IEMs such as glycine encephalopathy, creatine deficiency syndromes, maple syrup urine disease (leucine peak), and mitochondriopathies (elevated lactate). The presence of cortical dysgenesis or malformation of cortical development should not preclude a metabolic etiology as some IEM, e.g., Zellweger syndrome, are associated with malformations of cortical development.

### 10.4. Genetic Testing

When a specific disorder is suspected based on the clinical phenotype and results of biochemical testing, molecular genetic testing for pathogenic mutations must be performed to confirm the diagnosis and aid in genetic counseling. Mutation testing may also be the first-line investigation in disorders where no characteristic metabolites are present or no diagnostic enzyme assays are available, e.g., mutation analysis of SLC25A22 for mitochondrial glutamate transporter deficiency.

If no diagnosis is reached, targeted next generation sequencing gene panels for epileptic encephalopathy should be considered. These can help identify whether a patient has a previously described pathogenic mutation in a gene associated with the suspected disease or may yield new information about a previously unreported variant. In a study examining the yield of genetic testing in epileptic encephalopathies, genetic causes were identified in 28% of the 110 patients in a series of patients with epileptic encephalopathies. Of these, 7% had inherited metabolic disorders that included pyridoxine-dependent epilepsy caused by *ALDH7A1*mutation, pyridox(am)ine-5-phosphate oxidase (PNPO) deficiency, Menkes disease, cobalamin G deficiency, MTHFR deficiency, glucose transporter 1 deficiency, glycine encephalopathy, and pyruvate dehydrogenase complex (PDHC) deficiency [80]. In this study, 45% of patients obtained a genetic diagnosis by targeted next-generation sequencing epileptic encephalopathy panels. All the patients with IEM in this series had either clinical (e.g., Menkes disease with brittle hair) or biochemical features (e.g., GLUT1 deficiency with low CSF glucose level) suggestive of the underlying disorder.

Next-generation sequencing technologies, using whole exome sequencing (WES), and whole genome sequencing (WGS) may be considered in patients in whom by the abovementioned diagnostic strategies have failed to establish a diagnosis. Preliminary data suggest that it may be a promising strategy in identifying new genes, and pathogenic mutations hitherto not described. Recently, Ngoh et al. reported two brothers who presented with early infantile clonic seizures, and then developed infantile spasms along with cerebellar hypoplasia/atrophy on MRI [80]. Using whole exome sequencing techniques, the children were found to be compound heterozygous for mutations in the gene encoding mitochondrial arginyl-tRNA synthetase 2 (*RARS2*). From a research perspective, these technologies are leading to an ever-increasing number of new genes where mutational changes are responsible for clinical phenotypes of disorders where genetic etiologies were suspected but not proven.

### 10.5. Tissue Biopsies

Invasive procedures such as skin and muscle biopsies should be considered if biochemical and genetic testing have proven unhelpful. Ultrastructural abnormalities in the skin and muscle biopsy may yield diagnostic clues [81]. Biochemical assays of the respiratory chain enzymes in fresh muscle are needed to diagnose respiratory chain defects. Specific enzyme assays can be carried out in fibroblast cultures [82]. These studies have to be employed in consultation with biochemical geneticists and specialized clinics in tertiary and quaternary care.

## 11. Treatment

Specific treatments, where available, have been mentioned in the sections on the individual disorders above and summarized in Table 6. A sequential therapeutic trial with vitamin B6, folinic acid, and pyridoxal phosphate can be initiated in all infants with early onset treatment resistant seizures while specific assays for biochemical markers are underway. Biotin should be added when refractory seizures are seen in association with alopecia and seborrheic dermatitis. Thiamine supplementation (200–300 mg/day) may be considered in children with unexplained status epilepticus and brain imaging studies showing cortical and medial thalami hyperintensities in T2 weighted sequences, as these findings could be suggestive of thiamine transporter deficiency due to SLC19A3 mutations. Thiamine may also be beneficial for seizures in late-onset pyruvate dehydrogenase complex deficiency and thiamine-responsive variants of MSUD [13].

A ketogenic diet is the treatment of choice for GLUT1 deficiency. The ketogenic diet is also useful for pyruvate dehydrogenase deficiency and complex I mitochondripathies [83]. This diet is also useful in refractory epilepsy and super refractory status epilepticus, irrespective of etiology however, disorders that may worsen on starting the ketogenic diet must be excluded first. These include β-oxidation defects, disorders of ketolysis, and gluconeogenesis [84].

For disorders where no specific treatments exist, symptomatic treatment with conventional anti-epileptic drugs can be used in most patients, with some exceptions. Valproate use may cause fatal hepatotoxicity in patients with Alpers syndrome. Sodium valproate is generally contraindicated in mitochondrial disorders due to the risk of exacerbating the symptoms of mitochondrial disease, and in urea cycle defects (risk of hyperammonemia). Phenobarbital should be avoided in GLUT1 deficiency. Sodium channel blockers may worsen seizures in progressive myoclonic epilepsy.

Steroids are useful in many epileptic encephalopathies, but they must be used with caution as they alter metabolic pathways and could therefore be poorly tolerated. There have been reports of the use of steroids for the treatment of infantile spasms due to methylmalonic aciduria, mitochondrial disorders, atypical phenylketonuria, glutaric aciduria type 1 and isovaleric aciduria [13,66,73,85]. Newer treatments are on the horizon for many IEM that are orphan disorders. These may involve repurposing known molecules, developing fusion proteins that navigate across the blood/brain barrier, chaperones, gene and enzyme replacement therapies, which will no doubt offer new hope for presently untreatable conditions.

## 12. Conclusions

IEMs are a rare cause of epilepsy but seizures and epilepsy are common in IEM, where the central nervous system carries the principal burden of disease. Many of these disorders are amenable to specific treatments and hence timely and appropriate diagnosis is essential to prevent irreversible brain damage [13]. This requires knowledge about the clinical presentation, standardized vitamin trials, and availability of biochemical tests for diagnosis. IEMs that remain undiagnosed by clinical and biochemical testing, next-generation sequencing with gene panels, and whole exome sequencing may increase the diagnostic yield.

## Figures and Tables

**Table 1:** Classification of epilepsies associated with IEM according to the age at presentation.

Neonatal Period–Early Infancy	Late Infancy–Childhood	Adolescence–Adulthood
Pyridoxine-dependent epilepsy PNPO deficiency Folinic-acid responsive seizures Biotinidase deficiency Holocarboxylase synthetase deficiency GLUT-1 (Glucose transporter-1) deficiency Serine biosynthesis defects Molybdenum cofactor and Sulfite oxidase deficiency Menkes disease Non-ketotic hyperglycinemia Organic acidemias Urea cycle defects Peroxisomal disorders Congenital disorder of glycosylation Congenital and early infantile Neuronal ceroid lipofuscinosis (NCL) Mitochondrial disorders	Creatine synthesis defects Late infantile NCL Mitochondrial disorders Organic acidurias Sialidosis Gangliosidosis GLUT-1 deficiency Congenital disorders of glycosylation Purine metabolism defects Uridine responsive epilepsy caused by CAD mutations Disorders of methylation and folate metabolism Neurotransmitter defects Congenital disorders of autophagy	Juvenile NCL Lafora body disease and Unverricht–Lundborg disease Mitochondrial disorders: MELAS (Mitochondrial encephalomyopathy, lactic acidosis and stroke-like episodes), MERRF (Myoclonic epilepsy with ragged red fibers) Lysosomal storage diseases: Late onset gangliosidosis, Niemann–Pick type C, Gaucher type III GLUT-1 deficiency Porphyria Wilson’s disease

**Table 2:** Phenotypic presentations of epilepsy associated with IEM.

Clinical Presentation	Some Examples
Neonatal encephalopathy with seizures	Pyridoxine dependency epilepsy, PNPO deficiency, Non-ketotic hyperglycinemia, Maple syrup urine disease
Developmental delay with seizures	Menke’s kinky hair disease, biotinidase deficiency, organic aciduria, Smith–Lemli–Opitz syndrome, Serine deficiency disorders
Dysmorphic features, developmental delay, seizures	Zellweger syndrome, pyruvate dehydrogenase deficiency, congenital disorders of autophagy, Neu–Lavoxa syndrome
Neurodegenerative disorder with seizures	Neuronal ceroid lipofuscinosis, Tay–Sachs disease, Lafora body disease, GM1 gangliosidosis, Niemann–Pick disease
Epilepsy with movement disorders	Glucose transporter defect, Neurotransmitter disorders
Idiopathic epilepsy (otherwise normal child)	Glucose transporter defect

**Table 3:** Clinical features of neuronal ceroid lipofuscinosis.

Variable	Congenital	Early Infantile	Late Infantile	Juvenile
Gene	*Cathepsin D (CTSD*)	*PPT1* (*Palmitoyl-protein thioesterase 1*)	*TPP1* (*Tripepidyl Peptidase 1*)	*CLN3* (*Neuronal ceroid lipofuscinosis type 3*)
Age at onset	Before or around birth	6–24 months	2–4 years	4–10 years
Clinical features at presentation	Seizures, microcephaly	Regression of milestones, vision impairment, seizures	Cognitive decline, seizures, vision impairment	Early vision loss, seizures, cognitive decline

**Table 4:** Phenotypes of epilepsy associated with mitochondrial disease [66].

Type		Remarks
Catastrophic neonatal refractory⁄recurrent status epilepticus; epilepsia partialis continua; and myoclonic epilepsy.		Associated with refractory seizures and status, multi-organ failure and early death
Neonatal myoclonic epilepsy		Reported in Complex IV deficiency patients. Myoclonic seizures later evolve to infantile spasms, then becoming multifocal, with severe psychomotor delay and acquired microcephaly. EEG initially shows burst suppression, later evolution to hypsarrhythmia
Infantile spasms		Reported in patients with MT-AP6 (microtubule associated protein 6) mutation and of deficiencies of complexes I, IV, and II. Beginning at median age of 6 months
Refractory or recurrent status epilepticus		Majority of the patients have Alpers syndrome associated with *POLG* (Polymerase γ) mutations. Pre-existing psychomotor delay, onset of as status epilepticus consisting of generalized or unilateral clonic seizures starting at median 21 months of age, EEG shows posterior polyspike wave discharges, MRI shows cerebral and/or cerebellar atrophy, Variable degree of liver dysfunction noted
Epilepsia partialis continua		Epilepsy begins abruptly with focal clonic jerks, pre-existing cerebellar ataxia in some patients, Reported in patients with POLG1 mutations and defects in the Coenzyme Q (CoQ) metabolism pathway
Myoclonic epilepsy		Myoclonic seizures are the predominant symptom, reported in patients with mitochondrial DNA mutations, MRI shows basal ganglia/cerebellar atrophy
Focal epilepsy		Focal seizures, evolving to generalized seizures, arising from the occipital lobe in patients with MELAS

**Table 5:** Biochemical investigations in IEM.

Metabolite	Abnormality	Diagnosis
Blood		
Plasma amino acids	High glycine	Nonketotic hyperglycinemia (NKH)
	Low serine	Serine biosynthesis defects
	High phenylalanine	Phenylketonuria (PKU)
	High branched chain amino acids	Maple syrup urine disease (MSUD)
Uric acid	Low	Molybdenum cofactor deficiency
Copper and ceruloplasmin	Low	Menkes disease
Homocysteine	High	Methylene tetrahydrofolate reductase (MTFHR) deficiency, Disorders of vitamin B12 metabolism
Plasma VLCFA	High	Peroxisomal disorders
Isoelectric focusing of silaotransferrins	Abnormal transferrin glycoforms	Congenital disorders of glycosylation
Urine		
Organic acids	Specific organic acids	Organic acidemias (methylmalonic acid in methylmalonic aciduria and disorders of vitamin B12 metabolism
Sulfite and sulfocysteine	High	Sulfite oxidase and Molybdenum cofactor deficiency
Guanidinoacetic acid	High	GAMT deficiency
	Low	AGAT deficiency
Creatine	Low	GAMT and AGAT deficiency
	High	Creatine transporter deficiency
α-aminoadipic semialdehyde	High	Pyridoxine dependency, Sulfite oxidase and Molybdenum cofactor deficiency
CSF (with paired blood samples)		
Glucose	Low CSF-Blood glucose ratio (<0.4)	GLUT1 deficiency
Lactate	High	Mitochondriopathies
Amino acids	High glycine	Nonketotic hyperglycinemia
	Low serine	Serine biosynthesis defects
GABA	High	GABA transaminase deficiency
Glutamine	Low	Congenital glutamine deficiency
Pyridoxal 5′ phosphate	Low	PNPO deficiency, Pyridoxine-dependent epilepsy
Methylene tetrahydrofolate reductase	Low	MTFHR deficiency
Biogenic monoamine metabolites	Abnormalities in the levels of 3 *O*-methyldopa, l-Dopa, 5-hyroxytryptophan, 5-hydroxy-indole acetic acid, homovanillic acid, 3-hydroxy 4-methoxy propylglycol	Neurotransmitter disorders, homovanillic acid and 5-hydroxy-indole acetic acid low in PNPO deficiency and PDE
Methylation pathway metabolites	Low methionine	5 MTFHR deficiency, acquired or congenital cerebral folate deficiency, cerebral folate transporter defect

**Table 6:** Specific treatments for IEM associated with epilepsy [11].

IEM	Treatment
Pyridoxine dependency	Pyridoxine, folinic acid, lysine restriction, arginine supplementation
PNPO deficiency	Pyridoxal phosphate
Biotinidase deficiency and holocarboxylase synthetase deficiency	Biotin
GLUT1 deficiency	Ketogenic diet
Cerebral creatine deficiency	Creatine monohydrate, arginine restriction and ornithine supplementation in GAMT deficiency
Serine biosynthesis defects	Serine
Dihydrofolate reductase deficiency	Folinic acid
Folate receptor defect	Folinic acid
Disorders of CoQ10 biosynthesis	CoQ10
Menkes syndrome	Copper histidine
Molybdenum cofactor deficiency	Cyclic pyranopterin
Wilsons disease	Chelation with d-penicillamine, trientene or tetrathiomolybdate
Thiamine transporter deficiency due to mutations in *SLC19A3*	Thiamine
